# Length Polymorphisms in the Angiotensin I-Converting Enzyme Gene and the Serotonin-Transporter-Linked Polymorphic Region Constitute a Risk Haplotype for Depression in Patients with Coronary Artery Disease

**DOI:** 10.1007/s10528-020-09967-w

**Published:** 2020-05-05

**Authors:** Thomas Meyer, Isabel Rothe, Julia Staab, Hans-Christian Deter, Stella V. Fangauf, Stefanie Hamacher, Martin Hellmich, Jana Jünger, Karl-Heinz Ladwig, Matthias Michal, Katja Petrowski, Joram Ronel, Wolfgang Söllner, Cora Weber, Martina de Zwaan, Redford B. Williams, Christian Albus, Christoph Herrmann-Lingen, for the SPIRR-CAD Investigators

**Affiliations:** 1 Department of Psychosomatic Medicine and Psychotherapy, University of Göttingen Medical Center and German Center for Cardiovascular Research, Partner Site Göttingen, Göttingen, Germany; 2 Department of Psychosomatics and Psychotherapy, Charité Universitätsmedizin Berlin, Campus Benjamin Franklin, Berlin, Germany; 3 Institute of Medical Statistics and Computational Biology, Faculty of Medicine and University Hospital Cologne, University of Cologne, Cologne, Germany; 4 The German National Institute for State Examinations in Medicine, Pharmacy and Psychotherapy, Mainz, Germany; 5 Department of Psychosomatic Medicine and Psychotherapy, Klinikum Rechts der Isar, Technical University of Munich, Munich, Germany; 6 Institute of Epidemiology, Helmholtz Zentrum München, Munich, German Research Center for Environmental Health, Oberschleißheim, Germany; 7 Department of Psychosomatic Medicine and Psychotherapy, University Medical Center Mainz, Mainz, Germany; 8 Department of Psychotherapy and Psychosomatics, Technical University of Dresden, Dresden, Germany; 9 Medical Psychology and Medical Sociology, University Medical Center, University of Mainz, Mainz, Germany; 10 Department of Psychosomatic Medicine and Psychotherapy, Clinic Barmelweid, Barmelweid, Switzerland; 11 Department of Psychosomatic Medicine and Psychotherapy, Nuremberg General Hospital, Paracelsus Medical University, Nuremberg, Germany; 12 Department of Psychosomatic Medicine and Psychotherapy, Hannover Medical School, Hannover, Germany; 13 Department of Psychiatry and Behavioral Sciences, Duke University Medical Center, Durham, NC USA; 14 Department of Psychosomatics and Psychotherapy, University of Cologne, Cologne, Germany; 15 Klinik für Psychosomatische Medizin und Psychotherapie, Georg-August-Universität Göttingen, Waldweg 33, 37073 Göttingen, Germany

**Keywords:** Coronary artery disease, Depression, Genotyping, Haplotype, Prognosis

## Abstract

Genetic variations affecting the course of depressive symptoms in patients with coronary artery disease (CAD) have not yet been well studied. Therefore, we set out to investigate whether distinct haplotypes of the two insertion/deletion polymorphisms in the serotonin-transporter-linked polymorphic region (*5-HTTLPR*) and the angiotensin I-converting enzyme (*ACE*) gene located on chromosome 17 can be identified as risk factors for trajectories of depression. Clinical and genotyping data were derived from 507 depressed CAD patients participating in the randomized, controlled, multicenter Stepwise Psychotherapy Intervention for Reducing Risk in Coronary Artery Disease (SPIRR-CAD) trial, of whom the majority had an acute cardiac event before study inclusion. Depression scores on the Hospital Anxiety and Depression Scale (HADS) were assessed at baseline and at five follow-up time points up to 2 years after study entrance. At baseline, depression scores did not significantly differ between patients carrying the risk haplotype *ACE* D/D, *5-HTTLPR* I/I (*n* = 46) and the non-risk haplotypes (*n* = 461, 10.9 ± 2.7 versus 10.4 ± 2.5, *p* = 0.254). HADS-depression scores declined from study inclusion during the first year irrespective of the genotype. At each follow-up time point, HADS-depression scores were significantly higher in *ACE* D/D, *5-HTTLPR* I/I carriers than in their counterparts. Two years after study inclusion, the mean HADS depression score remained 1.8 points higher in patients with the risk haplotype as compared to subjects not carrying this haplotype (9.9 ± 4.2 versus 8.1 ± 4.0, *p* = 0.009). In summary, the presence of the *ACE* D/D, *5-HTTLPR* I/I haplotype may be a vulnerability factor for comorbid depressive symptoms in CAD patients.

## Introduction

In patients hospitalized for coronary events including acute myocardial infarction, depressive symptoms are common and have been linked to higher mortality (Lichtman et al. 2014). Latent class analysis has identified different long-term trajectories of depression in the context of comorbid coronary artery disease (CAD), both following an acute cardiac event and in stable disease (Kaptein et al. 2006; Martens et al. 2008; Romppel et al. 2012). In addition, numerous studies have implicated putative biomarkers in the development of CAD-associated comorbid depression, which have broadened our understanding of the underlying pathophysiological pathways. Novel techniques in the fields of transcriptomics, proteomics and genomics offer a growing platform for the identification of depression-associated biomarkers and may help to improve early diagnosis, including cases of comorbid conditions. However, compared to cross-sectional studies, clinical investigations with a longitudinal design addressing the impact of genetic variants for predicting trajectories of depressive symptoms are rare.

Genome-wide association studies and candidate gene studies identified specific genetic variants that may influence depressive symptoms in cardiac patients and help to decipher the mechanisms behind the disproportionately high prevalence of depressive symptoms among CAD patients (McCaffery et al. 2009). Notably, pathway analyses revealed a significant overlap of pleiotropic gene loci shared between depressive disorders and cardiovascular diseases (Amare et al. 2017). Recent advancements in identifying predictors of depression severity have focused primarily on pro-inflammatory biomarkers, including circulating cytokines, as well as neurotransmitters and key components in neurotrophic, neuroendocrine and metabolic pathways (Adibfar et al. 2016). Genetic variants significantly associated with depressive symptoms in CAD patients include mutations in genes resulting in impaired functioning of the serotoninergic system (Otte et al. 2007; Kim et al. 2015, 2017).

An insertion/deletion (I/D) polymorphism in the gene encoding the serotonin transporter, also known as solute carrier family 6 member 4 (*SLC6A4*), located in the serotonin-transporter-linked polymorphic region (*5-HTTLPR*), has been reported to moderate the influence of stressful life events on depression (Caspi et al. 2003). In relation to stressful life events, individuals with one or two copies of the short allele exhibited more depressive symptoms and suicidality than subjects homozygous for the long allele. This observation was confirmed by Kang et al. who demonstrated that the short allele may cause a genetic predisposition toward suicidal ideation in patients 2 weeks after an acute coronary syndrome (Kang et al. 2017). While a meta-analysis found no evidence of a strong interaction between stress and the *5-HTTLPR* genotype contributing to the development of depression (Culverhouse et al. 2018), one study demonstrated effects of environmental stress and gender on associations among depressive symptoms and the *5-HTTLPR* genotype (Brummett et al. 2008a). Whereas in males the I allele combined with a chronic stressor was linked to higher depression scores as compared to those in the non-stressor group and those with the D allele, in females the D allele may increase an individual’s susceptibility to depression under stressful life conditions. In a related study, Brummett et al. (2008b) found that tryptophan infusion resulted in a larger increase in depressive symptoms in males carrying the I allele and in women carrying the D allele.

A meta-analysis suggested that an I/D polymorphism in the angiotensin I-converting enzyme (*ACE*) gene, which accounted for nearly half the variance of the total phenotypic serum concentration (Rigat et al. 1990), displayed a modest association with CAD (Zintzaras et al. 2008). Furthermore, one report suggested that this length-variation polymorphism may influence therapeutic outcome in patients suffering from unipolar major depression with D allele carriers having shorter duration of hospitalization and more frequent remission (Baghai et al. 2001). However, longitudinal data from studies in stable CAD patients with moderate symptoms of depression are not available. Therefore, in this study we used genotyping data from a prospective, multicenter trial to evaluate the association between persisting depression and the two I/D polymorphisms in the *SLC6A4* and *ACE* gene located on chromosome 17.

## Methods

### Study Design and Participants

The study population consists of participants from the multicenter Stepwise Psychotherapy Intervention for Reducing Risk in Coronary Artery Disease (SPIRR-CAD) trial. This randomized, controlled study was designed to assess the effect of individual and group psychotherapy on depressive symptoms among patients with stable CAD (Albus et al. 2011). In brief, German-speaking patients aged 18 to 75 years were eligible for the trial when they had a documented stenosis (> 50%) in a recently performed coronary angiogram and, in addition, were screened positive for depressive symptoms with a score higher than 7 on the respective subscale of the Hospital Anxiety and Depression Scale (HADS). The majority of patients were recruited from medical care centers and many of them had recently experienced an acute cardiac event (Herrmann-Lingen et al. 2016). Exclusion criteria were chronic inflammatory diseases, severe mental disorders (e.g., psychosis, addiction), acute suicidal tendencies, and major depressive episodes, as assessed with the structural clinical interview for DSM-IV (SCID), since it appeared unethical to randomize patients into the usual care condition without offering appropriate treatment. During the recruitment phase from November 2008 to April 2011, the patients were randomly assigned to one of two parallel study arms. In the control group, only one individual informative session was conducted after randomization, whereas in the intervention arm, study participants received 3–5 initial sessions of individual therapy, and if depressive symptoms persisted with an HADS-depression score ≥ 8, in total 25 sessions of a manualized, weekly group psychotherapy over a period of 10 months were offered. The study protocol did not interfere with medical treatment ensuring that all patients received routine cardiologic care. The SPIRR-CAD study protocol strictly recommended the administration of selective serotonin reuptake inhibitors (SSRIs), since this class of antidepressant drugs is currently considered the safest to use in CAD patients. In contrast, tricyclic or tetracyclic antidepressants were rarely prescribed in our sample, because they are known to elicit pro-arrhythmogenic and cardio-toxic effects. As control for manual adherence by the experienced psychotherapists, videotapes from the group therapy sessions were analyzed by the core facility, and in addition, regular feedback was given by local supervisors. The main outcome from the SPIRR-CAD trial demonstrated that, in the whole sample, psychotherapy was not significantly superior to usual care (Herrmann-Lingen et al. 2016). However, type D personality, a psychological construct characterized by the combination of two enduring personality traits, namely “negative affectivity” (i.e., dysphoria, anxious apprehension, and irritability) and “social inhibition” (i.e., lack of self-assurance and reticence in social interaction), was a significant predictor of psychotherapy success.

### Ethics Approval and Informed Consent

The trial was conducted in accordance with the Helsinki Declaration. All patients gave their written informed consent before being assigned to one of the two parallel treatment arms. The study protocol, including genetic assessment, was first approved on the 25th of October 2007 by the Ethics Commission of the Medical Faculty at the University of Göttingen (05/10/07) and subsequently by the local ethics commissions from all other nine participating centers. The study was registered in June 2008 under the registration number NCT00705965 at https://register.clinicaltrials.gov and in March 2008 at https://www.isrctn.com/ under the registration number ISRCTN76240576.

### Assessment of Depressive Symptoms

As the main diagnostic outcome assessment in this secondary analysis, depressive symptoms were monitored by means of the self-rated HADS questionnaire. This widely used psychometric instrument was originally developed to screen for self-reported anxiety and depression in non-psychiatric patients (Zigmond and Snaith 1983; Herrmann 1997; Bjelland et al. 2002). Seven of the 14 mixed multiple-choice items relate to the severity of depressive symptoms, and each question has a four-point response category allowing individually graded, alternative answers with scores ranging from zero to three. On its depression subscale, the HADS focuses particularly on anhedonia, dysphoria, and reduced drive, whereas physical symptoms of depression are intentionally avoided in the instrument to minimize the influence of physical symptoms of organic diseases confounding the depression subscale. A cut-off value of ≥ 8 for the depression subscale is usually considered abnormal, as recommended by the manual for the German version of the scale (Herrmann 1997). Study participants were monitored at baseline (t1) and after 1 month (after the individual therapy sessions, t2), 6 months (beginning of group therapy, t3), 12 months (during group therapy, t4), 18 months (end of treatment, primary endpoint, t5) and 2 years (≥ 6 months after the end of group therapy, t6). After adjusting for reversely scored items, all responses were summed to obtain the respective HADS-depression score at baseline and the follow-up time points.

### *5-HTTLPR* and *ACE* Genotyping

Genomic DNA was isolated from buffy coat samples using the FlexiGene DNA kit from Qiagen (Hilden, Germany), according to the manufacturer´s instructions. Genotyping of the I/D polymorphism in the *5-HTTLPR* region of the *SLC6A4* gene (rs25531) was performed using polymerase chain reaction (PCR) with the following primer pair: 5´-GGCGTTGCCGCTCTGAATGC-´3 and 5´-GAGGGACTGAGCTGGACAACCAC-´3 (Heils et al. 1996). The amplification yielded fragments of 484 bp and 528 bp. For genotyping of the length polymorphism in the *ACE* gene (rs4646994), a 190 bp (D allele) or 490 bp (I allele) fragment was amplified using the following two primers: 5´-CTGGAGACCACTCCCATCCTTTCT-´3 and 5´-GATGTGGCCATCACATTCGTCAGAT-´3 (Rigat et al. 1992; Glenn et al. 2009). Since the D allele was preferentially amplified in this reaction, each sample found to have the D/D genotype was subjected to a second round of an independent PCR amplification with the insertion-specific primer pair 5´-TGGGACCACAGCGCCCGCCACTAC-´3 and 5´-TCGCCAGCCCTCCCATGCCCATAA-´3 (Lindpaintner et al. 1995). In this second, confirmatory PCR only the I allele yielded a 335-bp amplicon, but not the homozygous DD allele. All PCRs were carried out in a total volume of 25 µl PCR buffer, containing 2 µl of isolated DNA, 2 mM of MgCl_2_, 0.32 mM desoxy-NTP mix, 0.8 µM of each specific primer, and 1.5 U of innuTaq DNA polymerase (Analytik Jena, Jena, Germany). The PCR protocol was run on a Biometra cycler and included a denaturation step at 95 °C for 4 min and 35 cycles of denaturation at 95 °C for 1 min, annealing at 63 °C for 1 min, and extension at 72 °C for 10 min. PCR products were separated using electrophoresis on 1.5% agarose gels supplemented with ethidium bromide and visualized by ultraviolet transillumination. All laboratory procedures and ratings were carried out under single-blind conditions. To avoid misclassification, two operators independently scored the genotypes.

### Statistical Analysis

Deviations from the Hardy–Weinberg equilibrium were calculated using *χ*^2^ test. For demographic and clinical variables, descriptive statistics between the different allele carriers were calculated. These data are presented as means and standard deviations for continuous variables or percentages for categorical variables. Differences between subgroups were assessed using *χ*^2^ test for categorical measures and *t* test for continuous measures. If data distribution was not normal, the Mann–Whitney *U* test was used. To assess the longitudinal association of genotype distribution and depression, a repeated-measures ANOVA was performed. The measures of depression at baseline and the five follow-up time points were entered as within-subject outcomes and genotype distribution as between-subjects factor. In models where Mauchly’s test of sphericity was significant, Greenhouse–Geisser corrected values are reported. Post-hoc tests for multiple comparisons between the groups of different genotypes and haplotypes were performed. Pairwise comparisons between the three genotypes (D/D, D/I, I/I) were performed using Fisher’s Least Significant Difference (LSD) method. A general linear model comparing the homozygous haplotype groups was calculated. *p* values below 0.05 were considered statistically significant. Because of the exploratory nature and the a priori hypothesis of this study, we did not adjust for multiple comparisons. All statistical analyses were performed using SPSS version 25 (IBM Corp., Armonk, NY, USA).

## Results

The allele distributions for the *5-HTTLPR* and *ACE* gene were both in Hardy–Weinberg equilibrium (*5-HTTLPR*: *p* = 0.291 and *ACE*: *p* = 0.941). As shown in Table 1, the genotype groups for the two length polymorphisms did not significantly differ with respect to gender, age, body mass index, and smoking habits. Likewise, the Charlson Comorbidity Index (CCI) and the numbers of comorbid diagnoses, including diabetes mellitus, hypertension, hyperuricemia, family history of myocardial infarction, and previous coronary artery bypass grafting (CABG), were similar among the three genotype groups for each polymorphism (Table 1). The lipid profile (low-density and high-density lipoproteins, total cholesterol and triglyceride) did not significantly differ between the genotype groups. Similarly, echocardiographic measures of atrial and ventricular diameters as well as ejection fraction showed no significant differences between the genotypes. Dropouts from the study (*n* = 135) were more likely to be younger (57.5 ± 10.1 vs. 59.7 ± 9.2 years, *p* = 0.022), had a reduced ejection fraction (47.0 ± 12.4 vs. 51.6 ± 12.9%, *p* = 0.040), and a higher HADS depression score at baseline (10.9 ± 2.7 vs. 10.3 ± 2.4, *p* = 0.020) than their counterparts with complete follow-up data. However, neither the frequencies of the *5-HTTLPR* (*p* = 0.292) and *ACE* genotype (*p* = 0.927) nor their combined haplotypes (*p* = 0.871) differed with respect to the completeness of the follow-up data.

**Table 1:** Baseline characteristics of the SPIRR-CAD study cohort by *5-HTTLPR* and *ACE* genotypes

	*5-HTTLPR* genotype				*ACE* genotype			
	D/D (*n* = 104)	I/D (*n* = 337)	I/I (*n* = 166)	*p* value	D/D (*n* = 140)	I/D (*n* = 252)	I/I (*n* = 115)	*p* value
Male gender	76.0	82.8	76.5	0.189	78.7	80.2	78.3	0.888
Age (years)	58.9 ± 8.9	59.0 ± 9.5	59.2 ± 9.9	0.949	58.2 ± 9.7	59.3 ± 9.6	59.4 ± 9.1	0.475
Body mass index (kg/m^2^)	28.0 ± 4.5	28.8 ± 4.9	28.1 ± 4.9	0.219	28.7 ± 5.2	28.2 ± 4.5	28.6 ± 5.0	0.650
NYHA class	1.76 ± 0.72	1.89 ± 0.76	1.79 ± 0.68	0.373	1.78 ± 0.70	1.84 ± 0.75	1.87 ± 0.71	0.786
CCS angina class	2.27 ± 1.22	2.28 ± 1.15	2.29 ± 1.12	0.986	2.31 ± 1.57	2.26 ± 1.53	2.3 ± 1.16	0.887
Charlson Comorbidity Index (CCI)	2.14 ± 1.69	2.24 ± 1.45	2.05 ± 1.40	0.446	2.18 ± 1.22	2.12 ± 1.12	2.20 ± 1.29	0.870
Diabetes mellitus	20.8	28.0	22.2	0.257	21.3	26.1	25.4	0.568
Hypertension	86.1	91.6	88.4	0.290	90.5	89.6	87.7	0.765
Hyperlipidemia	88.1	88.5	89.7	0.905	89.9	88.4	88.6	0.901
Hyperuricemia	15.1	18.2	15.5	0.731	18.0	18.2	11.5	0.297
Family history of MI	24.3	34.7	35.0	0.266	30.7	35.7	28.3	0.557
Previous CABG	13.6	20.0	15.7	0.286	13.7	17.1	22.1	0.209
Smoking habits (smoker)	35.0	36.0	28.9	0.313	35.0	29.2	40.9	0.082
Antidepressant use	11.5	11.7	10.8	0.962	12.8	11.5	9.6	0.724
LDL (mg/dl)	101.2 ± 51.8	99.4 ± 50.2	106.4 ± 57.8	0.500	106.4 ± 46.8	102.1 ± 53.9	96.7 ± 58.2	0.475
Cholesterol (mg/dl)	169.6 ± 72.0	168.6 ± 68.4	173.6 ± 76.9	0.834	170.7 ± 72.2	160.1 ± 80.4	170.4 ± 71.8	0.231
HDL (mg/dl)	39.7 ± 18.7	40.0 ± 17.8	41.1 ± 19.7	0.846	42.3 ± 18.7	40.0 ± 17.4	38.4 ± 20.9	0.352
Triglyceride (mg/dl)	147 ± 122	151 ± 162	162 ± 140	0.608	154 ± 109	162 ± 133	136 ± 101	0.249
LVDed (mm)	49.4 ± 8.0	52.7 ± 8.2	49.3 ± 8.9	0.076	51.9 ± 7.0	51.1 ± 10.1	49.7 ± 5.7	0.584
LVEes (mm)	34.2 ± 8.3	36.8 ± 9.5	35.9 ± 7.9	0.564	37.1 ± 8.6	36.2 ± 9.8	33.9 ± 6.8	0.469
LA (mm)	40.4 ± 6.0	42.4 ± 6.6	42.4 ± 5.6	0.348	43.1 ± 6.0	41.5 ± 6.2	41.4 ± 6.7	0.378
Ejection fraction (%)	52.7 ± 11.7	49.5 ± 13.3	49.8 ± 12.7	0.509	50.0 ± 13.0	49.7 ± 12.4	51.6 ± 14.0	0.776
HADS anxiety baseline	10.3 ± 3.7	10.5 ± 3.8	10.5 ± 3.7	0.866	10.6 ± 3.7	10.2 ± 3.7	10.5 ± 3.7	0.550

Data are presented as mean ± standard deviation or percentage, respectively

*ACE* angiotensin I-converting enzyme, *CABG* coronary artery bypass grafting, *CCS* Canadian Cardiovascular Society, *HADS* Hospital Anxiety and Depression Scale, *HDL* high-density lipoprotein, *5-HTTLPR* serotonin-transporter-linked polymorphic region, *LA* left atrium, *LDL* low-density lipoprotein, *LVDed* left-ventricular end-diastolic dimension, *LVDes* left-ventricular end-systolic dimension, *MI* myocardial infarction, *NHYA* New York Heart Association, *SPIRR-CAD* Stepwise Psychotherapy Intervention for Reducing Risk in Coronary Artery Disease study

Data showed that the mean HADS-depression score in the total study population decreased successively from baseline (10.4 ± 2.5, *n* = 507) to 18-month follow-up (8.1 ± 3.9, *n* = 378), but was stable at 24-month follow-up (8.3 ± 4.0, *n* = 372) (Table 2A). This decline in self-rated depressive symptoms from study inclusion during the first 18 months was observed in all patient groups, regardless of the presence of the long or short allele for the *5-HTTLPR* and *ACE* polymorphism, respectively (Table 2A and B).

**Table 2:** HADS-depression scores at baseline (t1), after 1 month (t2), 6 months (t3), 12 months (t4), 18 months (t5) and 2 years (t6) by the *5-HTTLPR* (A) and *ACE* (B) genotype

A	Total study population	*5-HTTLPR* D/D	*5-HTTLPR* I/D	*5-HTTLPR* I/I	*p* value
HADS depression t1	10.4 ± 2.5 (*n* = 507)	10.6 ± 2.8 (*n* = 104)	10.3 ± 2.3 (*n* = 237)	10.5 ± 2.7 (*n* = 166)	0.425
HADS depression t2	9.8 ± 3.9 (*n* = 452)	10.2 ± 4.2 (*n* = 90)	9.6 ± 3.7 (*n* = 207)	9.9 ± 4.0 (*n* = 155)	0.494
HADS depression t3	8.9 ± 3.9 (*n* = 420)	9.3 ± 4.4 (*n* = 84)	8.6 ± 3.8 (*n* = 193)	9.2 ± 3.6 (*n* = 143)	0.247
HADS depression t4	8.6 ± 4.0 (*n* = 394)	8.6 ± 4.4 (*n* = 81)	8.3 ± 3.8 (*n* = 173)	8.9 ± 4.1 (*n* = 140)	0.398
HADS depression t5	8.1 ± 3.9 (*n* = 378)	7.9 ± 4.1 (*n* = 72)	7.9 ± 3.8 (*n* = 172)	8.4 ± 3.8 (*n* = 134)	0.516
HADS depression t6	8.3 ± 4.0 (*n* = 372)	7.7 ± 4.2 (*n* = 73)	8.4 ± 4.0 (*n* = 170)	8.4 ± 3.9 (*n* = 129)	0.379

B	*ACE* D/D	*ACE* I/D	*ACE* I/I	*p* value
HADS depression t1	10.6 ± 2.6 (*n* = 140)	10.5 ± 2.6 (*n* = 252)	10.1 ± 2.4 (*n* = 115)	0.317
HADS depression t2	10.3 ± 3.9 (*n* = 125)	9.6 ± 4.0 (*n* = 224)	9.7 ± 3.8 (*n* = 103)	0.277
HADS depression t3	9.5 ± 3.9 (*n* = 114)	8.8 ± 3.9 (*n* = 208)	8.6 ± 3.9 (*n* = 98)	0.217
HADS depression t4	9.3 ± 4.2 (*n* = 109)	8.4 ± 3.9 (*n* = 201)	8.2 ± 4.0 (*n* = 84)	0.087
HADS depression t5	8.5 ± 4.1 (*n* = 102)	8.0 ± 3.9 (*n* = 190)	7.9 ± 3.6 (*n* = 86)	0.527
HADS depression t6	9.0 ± 4.1 (*n* = 102)	8.2 ± 4.1 (*n* = 184)	7.6 ± 3.6 (*n* = 86)	**0.047**

Bold *p* values are less than 0.05

**Fig. 1:**
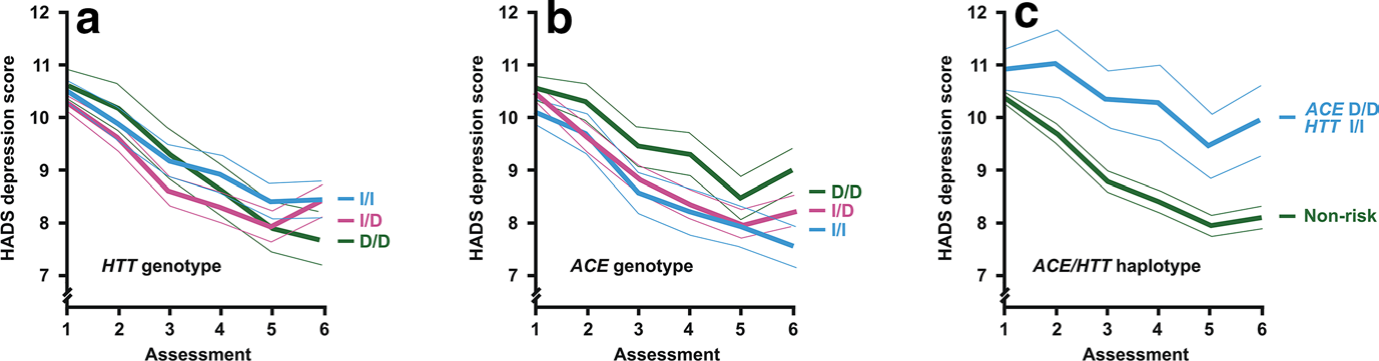
Trajectories of HADS depression scores in depressed coronary artery disease patients by genotypes of length polymorphisms in the serotonin-transporter-linked polymorphic region (*HTT*) (**a**) and the angiotensin I-converting enzyme (*ACE*) gene (**b**) as well as the risk haplotype (*ACE* D/D, *HTT* I/I) thereof (**c**). The graphs depict the means and the standard errors of the mean at baseline and five subsequent follow-up time points

The mean depression score 2 years after study inclusion was highest in homo- (8.4 ± 3.9) and heterozygous carriers (8.4 ± 4.0) of the *5-HTTLPR* insertion fragment and lowest in homozygous carriers of the deletion fragment (7.7 ± 4.2), but this difference among the genotypes was not statistically significant (*p* = 0.379) (Fig. 1a and Table 2A). For the *ACE* polymorphism rs4646994, ANOVA showed different mean HADS-depression scores among the three genotypes at 24-month follow-up. The mean depression score was highest in the group with the homozygous deletion of the gene fragment (9.0 ± 4.1) and lowest in carriers of the *ACE* I/I genotype (7.6 ± 3.6), while the heterozygous group displayed an intermediate depression score (8.2 ± 4.1) (Fig. 1b and Table 2B). This difference was statistically significant (*p* = 0.047) and suggested a gene-dosage effect. A similar result was obtained when the group of *ACE* D/D carriers was compared to the combined group of non-D/D genotype carriers (*p* = 0.034).

Carriers of the *ACE* D/D, 5-*HTTLPR* I/I haplotype had the highest HADS-depression scores at 24-month follow-up (9.9 ± 4.1, *n* = 36) in comparison to the other three homozygous genotype groups (*p* = 0.009, Fig. 1c) and also to all study participants not carrying this risk haplotype (8.1 ± 4.0, *n* = 336, *p* = 0.009, Table 3). However, sociodemographic and clinical data at baseline, including HADS depression and somatic comorbidities, did not differ between participants who carried the risk haplotype versus the remaining participants not carrying this haplotype. In particular, there was no significant link between the risk and non-risk haplotypes with respect to randomization to the intervention arm (58.7 vs. 49.5%, *p* = 0.280). Notably, patients with the risk haplotype differed significantly from all other patients with regard to depression score already at 1-month follow-up and at each later time point (*p* ≤ 0.032, Table 3). This difference was particularly evident in male study participants at 12-month (*n* = 29, 10.1 ± 4.7 vs. *n* = 286, 8.4 ± 3.9, *p* = 0.030) and 24-month follow-up, respectively (*n* = 29, 10.4 ± 4.4 vs. *n* = 274, 8.2 ± 3.9, *p* = 0.008). In females carrying the risk haplotype, the HADS-depression score was higher only at 18-month follow-up (*n* = 10, 10.4 ± 4.2 vs. *n* = 66, 7.6 ± 4.0, *p* = 0.043).

**Table 3:** Changes in HADS-depression scores from baseline to 2-year follow-up by the risk haplotype *ACE* D/D, *5-HTTLPR* I/I and the combined non-risk haplotypes. Patients were assessed at baseline (t1), after 1 month (t2), 6 months (t3), 12 months (t4), 18 months (t5) and 2 years (t6)

	Risk haplotype ACE D/D, *5-HTTLPR* I/I	Combined non-risk haplotypes	*p* value
HADS depression baseline t1 (*n* = 136)	10.9 ± 2.7 (*n* = 46)	10.4 ± 2.5 (*n* = 461)	0.254
HADS depression t2 (*n* = 126)	11.0 ± 4.3 (*n* = 44)	9.7 ± 3.9 (*n* = 408)	**0.032**
HADS depression t3 (*n* = 114)	10.3 ± 3.5 (*n* = 41)	8.8 ± 3.9 (*n* = 379)	**0.015**
HADS depression t4 *(n* = 107)	10.3 ± 4.5 (*n* = 39)	8.4 ± 3.9 (*n* = 355)	**0.006**
HADS depression t5 (*n* = 101)	9.5 ± 3.8 (*n* = 37)	8.0 ± 3.9 (*n* = 341)	**0.024**
HADS depression t6 (*n* = 102)	9.9 ± 4.1 (*n* = 36)	8.1 ± 4.0 (*n* = 336)	**0.009**

Bold *p* values are less than 0.05

A general linear model comparing the homozygous haplotype groups confirmed that the HADS-depression score differed significantly between the time points (*p* < 0.001). In addition, there was a significant between-subjects effect for the four haplotype groups considered separately (*p* = 0.013). When comparing the risk *ACE* D/D, *5-HTTLPR* I/I haplotype against the combined group of participants not carrying this haplotype, the difference in between-subjects effect was even more pronounced (*p* = 0.009). Repeated-measures ANOVA revealed a significant effect of time (*p* < 0.001), but no significant interaction term between time and risk genotype (*p* = 0.416).

## Discussion

This genetic study assessed the associations between two common functional polymorphisms in genes expressed in the central nervous system and trajectories of depressive symptoms in moderately depressed CAD patients. The length polymorphisms in the serotonin transporter (*SERT*) and the *ACE* gene are both located in non-coding gene regions and known to modulate the expression rate of their corresponding gene products. While the presence of the short variant in the *5-HTTLPR* region results in reduced mRNA transcription and diminished SERT synthesis as compared to its long allelic variant (Lesch et al. 1996), the *ACE* D allele is linked to higher serum ACE activities and elevated substance P concentrations in the brain (Rigat et al. 1990; Arinami et al. 1996). Using data from the randomized, multicenter SPIRR-CAD trial in CAD patients presenting with moderate depression, we confirmed that the severity of depressive symptoms generally declined up to 18 months and remained stable thereafter. According to the study protocol, the participants presented with elevated depression scores upon entrance into the study and many of them had experienced an acute cardiac event. Typically, the HADS-depression score constantly decreased during the first one and a half year. The main findings of our study indicate that there was a time-dependent decline in depressive symptomatology from study inclusion to 2-year follow-up and that the persistence of depressive symptoms was linked to genetic variations in the *ACE* and *5-HTT* genes, as carriers of the *ACE* D/D, *5-HTTLPR* I/I haplotype had significantly higher self-rated depression scores after 2 years as compared to the other haplotype carriers. Our data are in line with two studies by Brummett et al. demonstrating gender-specific effects of psychosocial stressors and tryptophan infusion on associations among symptoms of depression and the *5-HTTLPR* genotype (Brummett et al. 2008a, b). Also in our analysis, male carriers of the risk haplotype, which included the homozygous *5-HTTLPR I/I* alleles, had a significantly higher risk for persisting depressive symptoms at 12- and 24-month follow-up.

While the majority of genetic studies have investigated the relationship of each length polymorphism independently of the diagnosis of depression, in the present paper, we tested the hypothesis that the long-term outcome of depressive symptomatology differed among the *ACE*, *5-HTTLPR* haplotypes. In an early and important paper, Caspi and colleagues reported that subjects with one or two copies of the short D allele of the *5-HTTLPR* promoter polymorphism exhibited more depressive symptoms and suicidality in relation to stressful life events than individuals homozygous for the long I allele (Caspi et al. 2003). Cross-sectional data from the Heart and Soul Study confirmed that CAD patients carrying the D allele of the *5-HTTLPR* polymorphism were more vulnerable to depression, had a higher score for perceived stress, and an increased 24-h urinary norepinephrine secretion (Otte et al. 2007). Among low-income African American women, Cicchetti et al. showed that changes in depressive symptoms over time depended on the intervention group as well as the *5-HTTLPR* genotype, which in post-hoc analysis were indicative of differential susceptibility to interpersonal psychotherapy (Cicchetti et al. 2015). Studies showed a better response to selective serotonin reuptake inhibitor (SSRI) efficacy in I/I homozygous patients suffering from depression as compared to patients homozygous for the D allele who had a higher risk of not reaching remission (Yu et al. 2002; Arias et al. 2003; Hinkelmann et al. 2010).

While *5-HTTLPR* I/I-carriers exhibit increased 5-hydroxytryptamine clearance (Lesch et al. 1996), one report showed that this genotype was associated with smaller hippocampal volumes in patients with major depression but not in health controls (Frodl et al. 2004). However, a recently published collaborative meta-analysis based on multiple datasets found no evidence of a strong interaction between stress exposure and genetic variation in the *5-HTTLPR* locus which might contribute to the development of depression (Culverhouse et al. 2018). The failure of genome-wide association studies (GWAS) to identify candidate genes as risk factors for the development of major depression emphasizes that depression as a clinical diagnosis represents a genetically and nosologically heterogeneous spectrum of entities.

Data from the randomized, controlled Living Well With Stroke (LWWS) study in 101 depressed patients with ischemic stroke demonstrated that, opposite to the effects of antidepressant drug treatment with SSRIs, the psychosocial LWWS psychotherapy intervention was most effective in *5-HTTLPR* D allele carriers (Kohen et al. 2011). Since the D allele carriers benefited most from an interactive treatment supplying useful cognitive tools to enhance personal psychological resources, the LWWS investigators suggested that the presence of the D/D genotype may confer an increased sensitivity toward the social environment. This psychological component may also be important for the remission of depressive symptoms in our study participants from the SPIRR-CAD trial.

Using magnetic resonance imaging, Kobiella et al. reported that, during processing of unpleasant stimuli, healthy carriers of the short *5-HTTLPR* allele showed increased amygdala activation (Kobiella et al. 2011). The authors demonstrated that smaller volumes were associated with augmented amygdala activation and that D allele carriers displayed smaller amygdala volumes than the I/I genotype carriers. However, other studies did not reproduce this relationship between the *5-HTTLPR* polymorphism and baseline brain perfusion in the amygdala (Viviani et al. 2010). One study assessed the *5-HTTLPR* promoter polymorphism on the efficacy of a 1-week intensive exposure-based therapy for agoraphobia in a sample of patients with panic disorder and agoraphobia (Knuts et al. 2014). The low-expression D/D genotype showed a more favorable response to specific treatment compared to the other patients, which is in line with our results in depressed CAD patients.

Conflicting results have been published about the contribution of the *ACE* polymorphism as a risk factor for depression and suicidal behavior (Fudalej et al. 2009; Hishimoto et al. 2006; Sparks et al. 2009). Arinami et al. reported that in Japanese patients, the *ACE* D allele and DD genotype frequencies were significantly higher in patients with affective disorders than in controls (Arinami et al. 1996). However, this observation could not be confirmed in an independent German sample including 169 patients suffering from either bipolar disorder or unipolar recurrent major depression (Pauls et al. 2000). A few studies showed that patients with major depression carrying at least one D allele of the *ACE* I/D polymorphisms responded better to drug treatment than those who were homozygous for the I allele (Baghai et al. 2001; Bondy et al. 2005; Bahramali et al. 2016). However, a meta-analysis in 15 Chinese case-control studies showed no evidence of an association between the *ACE* I/D polymorphism and major depression including its response to treatment (Wu et al. 2012).

Annerbrink et al. (2010) assessed whether the *ACE* I/D polymorphism influenced the level of degradation products from monoamine neurotransmitters in the brain, since the ACE activity may impact on the intracerebral monoamine pathways. Based on the monoamine theory of depression, they measured the concentrations of 5-hydroxyindoleacetic acid (5-HIAA), which is the major metabolite of serotonin, and the dopamine metabolite homovanillic acid (HVA) in the cerebrospinal fluid (CSF) in healthy male subjects. The authors reported significant associations between the *ACE* genotypes and the CSF levels of 5-HIAA and HVA, while no relationship was found for the concentration of the noradrenaline metabolite 3-methoxy-4-hydroxyphenylglycol (MHPG). Furthermore, the authors replicated these observations in a small cohort of violent male offenders, suggesting a pathophysiologically relevant link between the *ACE* I/D polymorphism and unidentified components involved in the serotonergic and dopaminergic turnover. Given the functional relevance of ACE and the serotonin transporter for the modulation of the serotonergic pathway, it is not completely unexpected that genetic variations within these two genes may affect the outcome from depression, as suggested from our findings in a cohort of depressed CAD patients.

Important limitations of the current genetic study need to be addressed, as they relate to the fact that this is a clinical association study, as opposed to an experimental design with a direct manipulation. The analysis of genetic variations was not the primary purpose of the SPIRR-CAD trial; however, it was included a priori as a secondary research question. Due to the study protocol, we did not establish control groups with healthy subjects. Another limitation in this pilot study is that the clinical utility of our findings is questionable and remains to be determined. Furthermore, the number of dropouts was quite high, although the frequencies of genetic variants did not differ between participants with complete and incomplete follow-up data. The present findings are limited to Caucasian study participants and may not be generalized to other races or ethnicities. Finally, no independent and second study cohort was included for validation purposes. However, our investigation has also some strength which includes mainly a moderate-sized well-characterized study population with comprehensive clinical data. In addition, well-validated protocols for genotyping and psychometric assessment were used and high-standard quality checks were constantly performed. Future work should include attempts to directly replicate the present findings in multicenter trials, and if our findings can be confirmed, aimed at deciphering the underlying pathophysiological mechanisms.

In conclusion, we found that a haplotype of two previously documented functional length polymorphisms is associated with depressive symptoms in German patients with CAD. Our preliminary finding pointing to the *ACE* D/D, *5-HTTLPR* I/I haplotype as a factor for adverse outcome requires additional clinical and experimental investigations to extend our knowledge about the synergistic contribution of the serotoninergic and local renin–angiotensin system in the course of depression. If replicated in further research, this observation would suggest that depressed carriers of the risk haplotype may need specific treatments. This may be of particular relevance in depressed CAD patients who may require particular attention in secondary CAD prevention.
